# In Vitro Diagnostic Assays for COVID-19: Recent Advances and Emerging Trends

**DOI:** 10.3390/diagnostics10040202

**Published:** 2020-04-05

**Authors:** Sandeep Kumar Vashist

**Affiliations:** Pictor Private Limited, 24 Balfour Road Parnell, 1052 Auckland, New Zealand; sandeep.vashist@pictordx.com; Tel.: +49-177-7161461

**Keywords:** SARS-CoV-2, COVID-19, diagnostics, real-time reverse transcriptase polymerase chain (RT-PCR), lateral flow immunoassay (LFIA), immunoassays, point-of-care (POC)

## Abstract

There have been tremendous advances in in vitro diagnostic (IVD) assays for coronavirus disease 2019 (COVID-19) caused by severe acute respiratory syndrome coronavirus 2 (SARS-CoV-2). The main IVD assays used for COVID-19 employ real-time reverse transcriptase polymerase chain reaction (RT-PCR) that takes a few hours. But the assay duration has been shortened to 45 min by Cepheid. Of interest is the point-of-care (POC) molecular assay by Abbott that decreased the assay duration to just 5 min. Most molecular tests have been approved by the United States Food and Drug Administration (FDA) under emergency use authorization (EUA) and are Conformité Européenne (CE) marked. A wide range of serology immunoassays (IAs) have also been developed that complement the molecular assays for the diagnosis of COVID-19. The most prominent IAs are automated chemiluminescent IA (CLIA), manual ELISA, and rapid lateral flow IA (LFIA), which detect the immunoglobulin M (IgM) and immunoglobulin G (IgG) produced in persons in response to SARS-CoV-2 infection. The ongoing research efforts and advances in complementary technologies will pave the way to new POC IVD assays in the coming months. However, the performance of IVD assays needs to be critically evaluated before they are employed for the clinical diagnosis of COVID-19.

SARS-CoV-2 is a large positive-sense single-stranded ribonucleic acid (RNA) virus that comprises of four structural proteins, i.e., nucleocapsid protein (NP) that holds the viral RNA, spike protein (SP), envelope protein (EP), and membrane protein (MP), that create the viral envelope. It has a diameter of 50–200 nm and possesses spikes on its surface (up to 20 nm in length) that provide it the crown-like appearance, a characteristic of coronaviruses (CoVs). The lung disease caused by SARS-CoV-2 was given the name of COVID-19 by the World Health Organisation (WHO) on Feb 11, 2020. WHO declared COVID-19 as a public health emergency of international concern (PHEIC) on January 30, 2020 and a pandemic on March 11, 2020. Although China was the epicenter of the pandemic in the beginning, Europe was declared as the new epicenter on March 13, 2020 as the number of cases and deaths in Europe exceeded that of China. However, as the number of COVID-19 cases has increased significantly in the United States recently, it could become the new epicenter of COVID-19, as mentioned by the WHO on March 24, 2020.

Since its discovery in the Hubei province of Wuhan city in China in late December 2019, SARS-CoV-2 has now spread to 204 countries worldwide and also affecting two international conveyances, i.e., the Diamond Princess cruise ship, and the Holland America’s MS Zaandam cruise ship. The number of confirmed COVID-19 cases worldwide exceeds 1 million [1,2] while the number of global deaths exceeds 53,000. The United States of America (USA) has the most COVID-19 cases with over 245,000 cases, representing about 24% of the global COVID-19 cases. Italy, Spain, and Germany follow it, with over 115,000, 112,000, and 84,000 cases, respectively. However, Italy is the most affected country, as evident from the high mortality rate due to COVID-19 of over 14,000 deaths, while Spain and the USA are also seriously impacted with over 10,000 and 6000 deaths, respectively. The last few weeks have led to an exponential increase in the number of cases in the USA, UK, Iran, and the European Union (EU). However, Singapore, South Korea, and China have managed to successfully contain the spread of COVID-19 by taking all essential measures to deal with the pandemic.

SARS-CoV-2 was transmitted to humans via human–animal contact at the live animals market in Wuhan, China, in late December, 2019 [3]. Chinese researchers found its origin in bats, but the intermediate hosts for SARS-CoV-2 have not yet been identified [4]. The genomic sequence of SARS-CoV-2 has about 82% sequence homology to the human SARS-CoV and about 89% with bat SARS-like-CoVZXC21 [5]. The aged population (age above 65 years), and persons having decreased immunity or chronic diseases, such as hypertension, diabetes mellitus, cardiovascular diseases, and lung disease, are at a higher risk for developing severe COVID-19 if they are infected with SARS-CoV-2. Therefore, they must take special precautions to prevent the infection. The WHO has stressed the need for more intensive testing of suspected cases to identify COVID-19 infected people so that they can be quarantined in order to avoid the further spread of infection. It is also evident that the existing real-time reverse transcriptase polymerase chain (RT-PCR) assays are unable to detect the COVID-19 in the early stages of infection, and there are reports where it has given false negatives in subjects for up to two weeks [6,7]. The reason of false negative results by RT-PCR could be due to the improper extraction of nucleic acid from clinical materials and insufficient cellular material for detection. A chest computerized tomography (CT) scan act as a complementary diagnostic tool that enables physicians to effectively detect COVID-19 infection in several such RT-PCR false-negative cases [8,9]. Therefore, there is a need for developing better in vitro diagnostic (IVD) assays that can detect the SARS-CoV-2 infection reliably in persons even at the initial stage. The specific biomarkers involved during the early stage of COVID-19 infection should be investigated and employed for the development of diagnostics.

The structure of SARS-CoV-2 comprises of spikes, which are formed by the SP (Figure 1). SP is a major glycoprotein (Mol. Wt. ~180 kDa) that consists of two subunits, i.e., S1 and S2 [10]. S1 contains a receptor binding domain (RBD), which is responsible for recognizing and binding with the host cell receptor, i.e., angiotensin converting enzyme 2 receptor (ACE2) found in the lower respiratory tract [11]. On the other hand, S2 contains other basic elements needed for the membrane fusion. SP is the common target for neutralizing antibodies and vaccines, while the amino-terminal S1 subunit of SP is the most variable immunogenic antigen. Additionally, SARS-CoV-2 has NP (Mol. Wt. ~40 kDa), the most abundant viral phosphoprotein produced and shed during infection. The template mRNA of NP is the most abundant subgenomic RNA. NP exhibits high immunogenicity and can be detected in either serum or urine samples during the first two weeks of infection with peak viral shedding around ten days after infection [12]. Being a large protein, it can be detected via a sandwich immunoassay. Furthermore, SARS-CoV-2 contains MP, which is the most abundant protein on the complete virion particle. The EP is the smallest major structural protein of SARS-CoV-2, which is involved in viral assembly, release of virions, and pathogenesis.

Several serological immunoassays have been developed by IVD companies for the detection of SARS-CoV-2 viral proteins and antibodies in the serum or plasma. The most widely used biomarkers for the detection of SARS-CoV-2 infection in commercial immunoassays (rapid lateral flow immunoassay (LFIA) tests, automated chemiluminescence immunoassay (CLIA), manual ELISA, and other formats) are IgM and IgG antibodies produced in suspects from the 2nd week of viral infection. IgM can be detected in the patient samples from 10 to 30 days after SARS-CoV-2 infection, while IgG can be detected from 20 days onwards [13]. The IgM response occurs earlier than that of IgG, but it then decreases and disappears. On the other hand, IgG can persist after infection for a long time and may have a protective role. Several manual ELISA kits are also commercially available for the detection of NP and SP, but these are used mainly for research.

The most widely used IVD assays for the confirmatory diagnosis of COVID-19 are based on RT-PCR that have been employed globally to deal with the pandemic. A novel and robust real-time RT-PCR assay was developed by Tib-Molbiol, Germany, in collaboration with various partners by the 2nd week of January 2020 [14]. It was highly specific for SARS-CoV-2 RNA and did not cross-react with other coronaviruses. The test detects the SARS-CoV-2 RNA via envelope (E) and RNA-dependent RNA polymerase (RdRp) gene assays. The E-gene assay was used for first line screening, while the RdRp gene assay was employed for confirmatory testing. In another approach, the researchers developed one-step RT-PCR assays to detect ORF1b and N regions of SARS-CoV-2 in 1 h and 15 min [15]. The N gene assay was used for screening, while Orf1b was used as confirmatory test. However, as the assay involved the detection of ORF1b and N regions that are highly conserved among sarbecoviruses, it could also bind to SARS-CoV and other closely-related viruses. The authors mentioned that they could distinguish the SARS-CoV-2 from SARS-CoV via sequence analysis of positive amplicons if the RT-PCR results are positive. Of interest was the development of real-time RT-PCR assay for the detection of RdRp/helicase (H) genes of SARS-CoV-2, which did not cross-react with other human coronaviruses and respiratory viruses [16]. Due to its high sensitivity, it can be used for COVID-19 detection in samples that have low viral loads and for testing saliva, plasma, and upper respiratory tract samples. Many RT-PCR assays have already been developed by many companies and researchers worldwide. The most prominent real-time RT-PCR rapid test is the Xpert^®^ Xpress SARS-CoV-2 test by Cepheid, USA, which provides results in just 45 min using GenXpert benchtop system [17]. It is a rapid and automated point-of-care (POC) molecular test that enables the qualitative detection of SARS-CoV-2 in nasopharyngeal swab, nasal wash, or aspirate specimens from suspects. The test requires only a minute for sample preparation, employs Cepheid’s Xpert^®^ Xpress cartridge technology, and targets multiple regions of the viral genome. It has also received Food and Drug Administration (FDA) emergency use authorization (EUA). Another prominent advance is the Vivalytic COVID-19 test by Bosch, Germany [18], which detects SARS-CoV-2 infection in suspects in less than 2.5 h. The test was developed by Bosch in collaboration with Randox Laboratories, UK. It is a fully automated and rapid POC molecular test that can simultaneously detect SARS-CoV-2 and nine other respiratory viruses, including influenza A and B. The sample is taken from the nose or throat of suspect using a swab and placed inside a Vivalytic cartridge containing all the assay reagents. The cartridge is then plugged into a small benchtop Vivalytic analyzer.

Abbott ID Now™ COVID-19 test is the most recent breakthrough IVD assay that detects SARS-CoV-2 in just 5 min [19,20]. It is a molecular POC test that utilizes the isothermal nucleic acid amplification technology for the qualitative detection of viral RNA from SARS-CoV-2. The test can be used in any location, such as hospitals, clinics, physicians’ offices, or in outbreak hotspots of COVID-19. It requires just a portable touchscreen-operated instrument, i.e., ID Now, which is lightweight (6.6 pounds) and compact (the size of a small toaster). It employs a molecular test for the RdRp gene, and can take throat, nasal, nasopharyngeal and oropharyngeal swabs as samples. The kit contains 24 tests, positive and negative controls, swabs for sample collection, and pipettes. It has just received the FDA EUA and is being seen as a remarkable achievement worldwide.

Apart from the molecular diagnostics, numerous LFIA based rapid POC tests have been developed by several companies, which enable the detection of IgM and IgG antibodies produced in suspects in response to SARS-COV-2 infection. One of the most prominent rapid tests is the COVID-19 test developed by BioMedomics, USA, which detects IgM and IgG antibodies in suspects in just 10 min [21]. It requires minimal sample volume, i.e., 20 µL of finger-pricked blood or 10 µL of serum/plasma. It does not require any instrument or trained staff and, thus, it can be employed at any place and time, especially in developing nations with limited healthcare resources and remote settings. The assay is ideal for primary healthcare workers for the rapid testing of COVID-19 suspects. Another prospective test is the SARS-CoV-2 rapid by Pharmacyt AG, Germany [22], which employs only two drops of finger-pricked blood sample from the suspects and can provide results in 20 min. The results obtained by the rapid test correlated well with those achieved by RT-PCR. The most exciting advance is the DPP COVID-19 IgM/IgG test launched recently by Chembio Diagnostics, USA, which has already received FDA EUA. It is a POC rapid LFIA test that provides results in just 15 min using finger-pricked blood sample. However, the distinct feature of the test is that it does not rely on the visual detection by naked eye but employs optical readout via MicroReader 1 and 2 analyzers produced by Chembio, Germany [23]. A large number of rapid IgM/IgG tests have been developed by several IVD companies, such as Beijing Lepu Medical Technology, Sona Nanotech, Jiangsu Medomics Medical Technologies, Innovita Biological Technology, Guangzhou Wondfo Biotech, Zhejiang Orient Gene Biotech, Biomerica, Xiamen AmonMed Biotechnology, Sugentech, CHIL, Zhenjiang Orient Gene Biotech, Sure Bio-Tech. However, there are very few peer-reviewed publications [24] that show the accuracy of the COVID-19 diagnostic results obtained by the rapid tests with respect to RT-PCR tests. Rapid tests could be used as a complementary IVD assay to the existing RT-PCR assays, which could lead to much better diagnosis of COVID-19 and provide additional information about the immune status of the suspects. However, the clinical accuracy of rapid tests needs to be stringently evaluated before they are authorized for the mass screening of COVID-19. The recent reports from many European countries suggest that most of the rapid tests for COVID-19 procured from China did not show good analytical performance and did not work for over 70% of COVID-19 cases [25,26,27,28]. Furthermore, as IgM and IgG could only be detected in suspects about two weeks after the onset of infection, there is an immense need to include other biomarkers of the early stage of SARS-CoV-2 infection for developing improved COVID-19 rapid tests.

An exciting development is the DZ-Lite SARS-CoV-2 CLIA IgM and IgG tests developed by Diazyme, USA, that have received FDA EUA [29]. The tests are based on the principle of chemiluminescence immunoassay (CLIA) and run on an automated Diazyme DZ-Lite 3000 Plus chemiluminescence analyzer with a throughput of 50 tests/h. Similarly, Snibe, China, has developed automated CLIA tests on MAGLUMI CLIA analyzers for the detection of IgG and IgM in the patient sample in 30 min [30]. The main advantages of automated CLIA analyzers based COVID-19 assays compared to rapid LFIA tests is the very high throughput of samples that can be analyzed and the ability to perform more clinical tests for other biomarkers, such as C-reactive protein (CRP), which also need to be monitored in COVID-19 suspects.

Several CE marked manual ELISA kits have also been developed by various IVD manufacturers, such as Euroimmun, IBL International, DRG Diagnostics GmbH, and Epitope Diagnostics. Most of them target the IgG and IgM produced in subjects in response to SARS-CoV-2 infection.

Other prospective IVD assay formats, such as those based on reverse transcription loop-mediated isothermal amplification (RT-LAMP), lab-on-a-chip, microfluidics, and multiplex detection are currently being investigated by researchers. It would also be useful to develop an automated and fully integrated POC IVD assay that can perform molecular and immunological testing on a single analytical platform. An ideal IVD assay for the POC detection of COVID-19 in the near future would be the smartphone-based POC electrochemical test for SARS-CoV-2 biomarkers, similar to the iHealth Align device developed by iHealth, USA, for smartphone-based blood glucose monitoring [31]. Smartphones are omnipresent, powered by internal battery, and equipped with large storage capacity, advanced processing power and a large screen display. Moreover, they have global positioning system (GPS) for spatiotemporal tagging of data, internet connectivity, and capability to store the processed results in the internal device memory as well as to a secure Cloud. Therefore, smartphone-based POC electrochemical device for the detection of SARS-CoV-2 biomarkers could be very useful for the large-scale rapid testing of COVID-19 suspects at the point-of-need.

The accurate diagnosis of people infected with the SARS-CoV-2 is essential to curb the global spread of COVID-19. However, the current RT-PCR based diagnostic assays are not robust, as they are still missing several infected cases [6,8,9]. Moreover, they can only be performed in well-equipped central laboratories by highly skilled analysts. Therefore, they are of limited utility and cannot be deployed widely, such as in developing nations, remote locations, and regions with decentralized laboratories. The delay in diagnosing people until after they have passed the disease onto many others is contributing to the continued global spread of COVID-19. The rapid LFIA and automated CLIA tests for IgM and IgG could complement the existing COVID-19 testing by RT-PCR. However, there is a need to stringently evaluate the clinical performance of commercial tests before they are used for the diagnosis of COVID-19. The biomarker discovery could also play a key role in identifying novel biomarkers that are involved in the early stages of SARS-CoV-2 infection. The recently developed Abbott ID Now™ COVID-19 test that detects SARS-CoV-2 in 5 min is a remarkable achievement [19,20] and could revolutionize COVID-19 testing. The ongoing efforts will further lead to reliable, robust, rapid, and easy-to-implement IVD assays and smart diagnostics for COVID-19. There are still significant knowledge gaps in health research that are crucial for an effective public health response in the case of epidemics. It signifies the need for all nations to invest in health research and make it an integral part of healthcare.

## Figures and Tables

**Figure 1:**
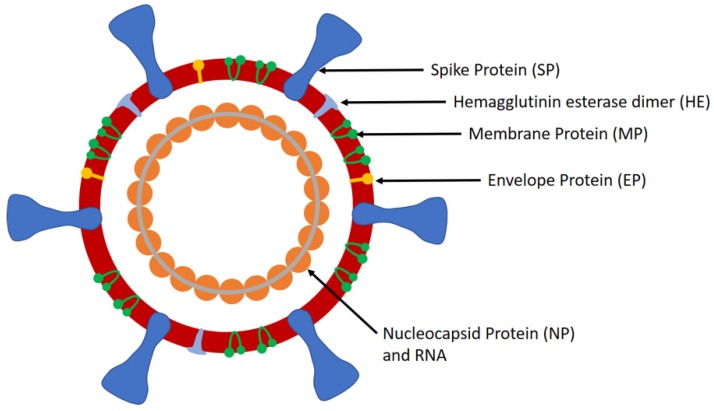
Schematic of the SARS-CoV-2.
